# Effect of exoskeletal joint constraint and passive resistance on metabolic energy expenditure: Implications for walking in paraplegia

**DOI:** 10.1371/journal.pone.0183125

**Published:** 2017-08-17

**Authors:** Sarah R. Chang, Rudi Kobetic, Ronald J. Triolo

**Affiliations:** 1 Department of Veterans Affairs, Advanced Platform Technology Center, Louis Stokes Cleveland Department of Veterans Affairs Medical Center, Cleveland, Ohio, United States of America; 2 Department of Biomedical Engineering, Case Western Reserve University, Cleveland, Ohio, United States of America; 3 Department of Orthopaedics, Case Western Reserve University, Cleveland, Ohio, United States of America; Semmelweis Egyetem, HUNGARY

## Abstract

An important consideration in the design of a practical system to restore walking in individuals with spinal cord injury is to minimize metabolic energy demand on the user. In this study, the effects of exoskeletal constraints on metabolic energy expenditure were evaluated in able-bodied volunteers to gain insight into the demands of walking with a hybrid neuroprosthesis after paralysis. The exoskeleton had a hydraulic mechanism to reciprocally couple hip flexion and extension, unlocked hydraulic stance controlled knee mechanisms, and ankles fixed at neutral by ankle-foot orthoses. These mechanisms added passive resistance to the hip (15 Nm) and knee (6 Nm) joints while the exoskeleton constrained joint motion to the sagittal plane. The average oxygen consumption when walking with the exoskeleton was 22.5 ± 3.4 ml O_2_/min/kg as compared to 11.7 ± 2.0 ml O_2_/min/kg when walking without the exoskeleton at a comparable speed. The heart rate and physiological cost index with the exoskeleton were at least 30% and 4.3 times higher, respectively, than walking without it. The maximum average speed achieved with the exoskeleton was 1.2 ± 0.2 m/s, at a cadence of 104 ± 11 steps/min, and step length of 70 ± 7 cm. Average peak hip joint angles (25 ± 7°) were within normal range, while average peak knee joint angles (40 ± 8°) were less than normal. Both hip and knee angular velocities were reduced with the exoskeleton as compared to normal. While the walking speed achieved with the exoskeleton could be sufficient for community ambulation, metabolic energy expenditure was significantly increased and unsustainable for such activities. This suggests that passive resistance, constraining leg motion to the sagittal plane, reciprocally coupling the hip joints, and weight of exoskeleton place considerable limitations on the utility of the device and need to be minimized in future designs of practical hybrid neuroprostheses for walking after paraplegia.

## Introduction

Stepping can be restored in individuals with paraplegia due to spinal cord injury (SCI) using technologies such as functional neuromuscular stimulation (FNS) [1,2], passive lower limb bracing most notably reciprocal gait orthoses (RGO) [3], powered exoskeletons [4], and hybrid neuroprostheses (HNP) [5,6].

Neural stimulation can provide the necessary joint torques required for standing and stepping by contracting the lower limb muscles in a coordinated manner [1,2]. Passive lower limb bracing can enable stepping by locking joints to support the user while the upper extremities balance and advance the body [3]. Powered exoskeletons have motors capable of supporting and moving lower extremity joints through the trajectories needed to complete steps [4]. In general, commercially-available powered exoskeletons (12–23 kg) have electric motors at hip and knee joints to move the lower limbs through a preset walking pattern. The HNP is a muscle-first approach that combines the primary power derived from FNS and the stability of passive lower limb controllable bracing (exoskeleton) [5,6]. Neural stimulation generates the necessary joint moments required for walking in paraplegia, while the exoskeleton provides the ability to support and stabilize the user by locking, unlocking, coordinating joints, and constraining degrees of freedom to the sagittal plane. Exoskeletons can be instrumented with sensors for feedback control to coordinate neural stimulation with context-dependent joint constraints. Most systems for walking in individuals with paraplegia require some upper body effort to maintain balance by means of walking aids. In addition, because muscle stimulation response differs among individuals and muscles fatigue rapidly at high duty cycle of stimulation required for walking, power assistance by external motors has recently been proposed and implemented to improve walking in hybrid systems [7,8,9].

Each of these technologies requires users to expend a certain amount of metabolic energy to walk that is typically more than required during able-bodied walking. Metabolic energy can be measured in the volume of oxygen consumed (ml of O_2_/min/kg). In addition, the physiological cost index (PCI) measured in heart beats per meter is often used to report energy cost of walking [10,11]. While this method is not reliable for comparison across subjects, it is reliable for comparing energy cost within a subject under different conditions [12].

Able-bodied individuals walking at an average normal speed of 1.3 m/s use about 3.4 times the average amount of oxygen consumed at rest [13], expressed as a “metabolic equivalent” where one MET is approximately 3.5 ml O_2_/min/kg. An individual with paraplegia uses 8 METs when walking at 0.5 m/s with FNS-alone [2] and 4.4 METs when walking with an RGO at approximately 0.2 m/s [5]. In comparison, individuals with paraplegia walking in powered exoskeletons had metabolic demands averaging 3.3 METs at an average speed of 0.27 m/s [14].

Only a limited number of muscles can be activated via stimulation for walking after paraplegia, resulting in generation of joint moments less than able-bodied values but adequate to restore stepping motions before onset of muscle fatigue [1]. Therefore, users of FNS-only systems rely substantially on the upper extremities for support and balance. As a result, the metabolic energy consumed when walking with stimulation alone or combined with an exoskeleton in a hybrid neuroprosthesis is considerably higher than for normal walking at comparable speeds. In order to isolate the effects of the exoskeleton and separate them from the metabolic costs of stimulation, only able-bodied subjects were evaluated in this study. Unimpaired walking by abled-bodied individuals provides the optimal neuromuscular system for stepping in the exoskeleton, since normal muscle activation is available. An exoskeleton that increases effort and limits walking speed in able-bodied individuals is also likely to hinder walking in individuals with paraplegia whose muscles may be deconditioned and easily fatigued with stimulation. Therefore, we hypothesized that able-bodied individuals walking with the joint constraints of an exoskeleton could attain speeds relevant for community ambulation at sustainable levels of metabolic energy expenditure. In this study, we evaluated the performance of the passive exoskeletal component of the HNP system during able-bodied walking, as measured by metabolic energy consumption, physiological cost index, walking speed, cadence, and step length.

## Methods

Six able-bodied individuals (Table 1) volunteered and signed the written informed consent forms to participate in this study approved by the Louis Stokes Cleveland Department of Veterans Affairs Medical Center Institutional Review Board. Data were collected within one week of enrollment over the course of a one-month period. Participants walked in the exoskeleton over level ground. The subjects were an average of 45 ± 17 years old, 67 ± 6 kg in weight, and 170 ± 6 cm in height.

**Table 1:** Subject characteristics.

Subject	Gender	Age (years)	Weight (kg)	Height (cm)
1	F	27	62	165
2	M	66	73	180
3	M	23	67	170
4	M	58	74	173
5	M	53	68	167
6	F	45	57	164

The joint constraints of the exoskeleton were implemented at the hip and knee by means of previously described hydraulic mechanisms [15,16,17]. The variable constraint hip mechanism can reciprocally couple, lock, or unlock the hip joints [15], and the stance control knee mechanism can lock or unlock the knee joints [16]. The ankle joints were locked at neutral. Average passive resistances to rotary motion at the hip and knee joints were determined for the mechanisms in bench testing with a dynamometer. During 1:1 hip reciprocal coupling evaluated at 120°/sec, passive resistance for hip flexion and extension was an average of 13.0 Nm and 17.1 Nm, respectively. The passive resistance at the knee during rotations at 120°/sec was 3.7 Nm and 3.6 Nm for knee flexion and extension, respectively.

**Fig 1:**
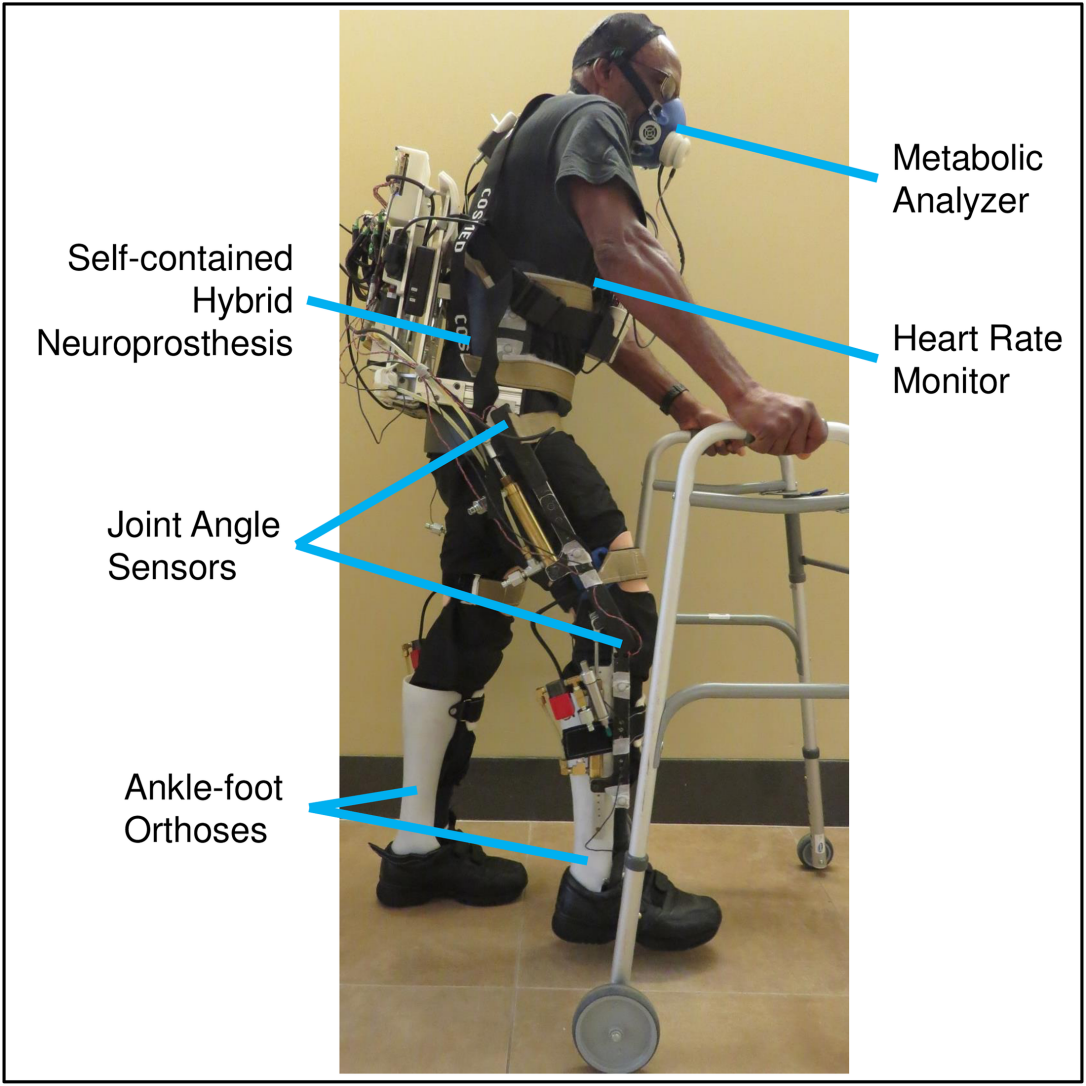
Subject wearing exoskeleton and metabolic analyzer.

The exoskeleton was setup by priming the hip and knee hydraulic circuits and adjusting the upright lengths for each participant such that the hip and knee joint centers were aligned [15,16]. The ankles were fixed at neutral in solid ankle-foot orthoses while the hips were reciprocally coupled and knees were free to move. Subjects were strapped into a thoracic corset attached to the pelvic band of the exoskeleton. Once the exoskeleton was donned while sitting in a chair at the end of the walkway, subjects practiced walking for approximately five minutes to ensure correct fit and alignment, and to familiarize themselves with stepping in the device. All subjects held onto a walker for safety. After the initial practice period, subjects returned to sitting, and a K4 b^2^ metabolic analyzer (COSMED, Rome, Italy) and heart rate monitor were donned (Fig 1). The individual in Fig 1 has given written informed consent (as outlined in PLOS consent form) to publish this image.

Baseline oxygen consumption was measured with subjects sitting at rest. After five minutes, the subjects were instructed to stand up and walk as fast as possible. The exoskeleton was set in reciprocal gait orthosis mode with the hips reciprocally coupled, the knees unlocked, and ankles locked at neutral. The subjects walked for approximately five minutes, four times down and back along a 95 m long hallway turning at each end. Oxygen consumption and heart rate were recorded on the metabolic analyzer and joint angle data (US Digital encoders, Vancouver, WA, USA) from the brace were recorded wirelessly on a laptop via Bluetooth. After four laps, the subjects returned to sitting for five minutes or until their heart rate and oxygen consumption returned back to resting values. The subjects then removed the exoskeleton and returned to sitting at rest before performing the walk again without the exoskeleton. After five minutes of sitting at rest, the subjects were instructed to stand up and repeat walking at a speed matching that previously attained with the exoskeleton, while oxygen consumption and heart rate data were recorded. Speed matching in the second condition of free walking without the exoskeleton was ensured by comparing the cadence and walking speed (number of steps and time) at regular intervals and verbally instructing the need to speed up or slow down to match the cadence and speed in the first condition of walking with the exoskeleton. After walking, the subjects returned to sitting at rest for approximately five minutes.

Number of steps, time to walk each length of 95 m, heart rate, and oxygen consumption were recorded. From these data, the physiological cost index (PCI), average walking speed, cadence, and step length were calculated. Oxygen consumption and heart rate were averaged over the last 2–3 minutes of walking when steady state was reached. The average PCI when walking with and without the exoskeleton was calculated by subtracting the average resting heart rate from the average walking heart rate and dividing by walking speed [12]. The data were analyzed using a one-way analysis of variance test (ANOVA) with Bonferroni multiple-comparison correction for a 95% confidence interval (p < 0.05) with Minitab 17 Statistical Software (Minitab Inc., State College, Pennsylvania) to determine the statistically significant differences between walking with and without the exoskeleton. Joint angles were recorded only when walking with the exoskeleton.

## Results

All subjects were able to achieve a walking speed appropriate for community ambulation [18,19,20] in the exoskeleton with hips coupled, knees free and ankles fixed at neutral. The average maximum walking speed with the exoskeleton across all subjects was 1.2 ± 0.2 m/s, with cadence of 104 ± 11 steps/min, and step length of 70 ± 7 cm. When normalized to height, the average step length was not significantly different within each subject between conditions. Without the exoskeleton, subjects walked with an average speed of 1.3 ± 0.2 m/s, cadence of 108 ± 26 steps/min, and step length of 73 ± 7 cm. Table 2 lists the gait parameters for each subject. These were not significantly different within subjects (p>0.05). However, when compared between subjects, step length for Subject 5 in the exoskeleton was significantly different (p<0.05) compared to Subject 2 walking with and without the exoskeleton. The average step length normalized to height was significantly different (p<0.05) between Subject 5 with the exoskeleton and Subject 1 without the exoskeleton.

**Table 2:** Average walking speed, cadence, and step length for each subject walking with and without the exoskeleton.

Subject	Speed [m/s]		Cadence [steps/min]		Step Length [cm]	
	With Exo	Without	With Exo	Without	With Exo	Without
1	0.9 ± 0.1	1.3 ± 0.4	86 ± 4	106 ± 50	66 ± 5	77 ± 10
2	1.4 ± 0.1	1.4 ± 0.4	103 ± 5	112 ± 47	80 ± 1	79 ± 11
3	1.2 ± 0.1	1.3 ± 0.2	97 ± 5	107 ± 12	71 ± 2	70 ± 3
4	1.2 ± 0.1	1.2 ± 0.1	105 ± 2	102 ± 5	68 ± 8	72 ± 3
5	1.2 ± 0.1	1.2 ± 0.03	117 ± 4	105 ± 1	63 ± 5	68 ± 2
6	1.4 ± 0.1	1.4 ± 0.1	113 ± 4	116 ± 5	73 ± 2	71 ± 2
**Average**	**1.2 ± 0.2**	**1.3 ± 0.2**	**104 ± 11**	**108 ± 26**	**70 ± 7**	**73 ± 7**

The average peak hip and knee angles when walking in the exoskeleton were 25 ± 7° and 40 ± 8°, respectively. The average peak hip angular velocity was 128 ± 16°/sec, with individual subject averages ranging from 117 to 142°/sec. The average peak knee angular velocity was 234 ± 70°/sec, with individual subject averages ranging from 169 to 328°/sec.

**Fig 2:**
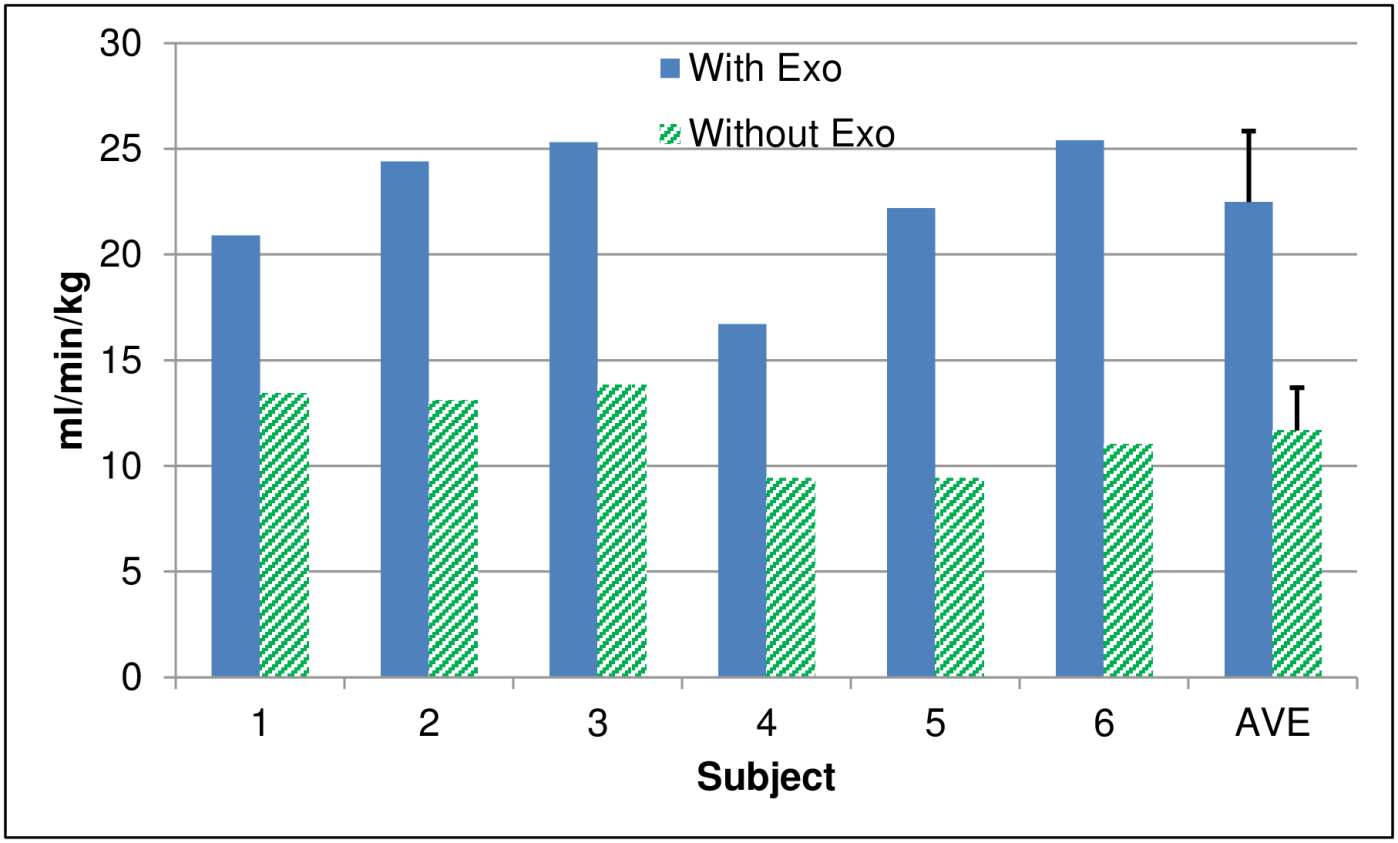
O_2_ consumption for each subject and averaged across all subjects when walking with and without the exoskeleton.

Oxygen consumption was 1.6 to 2.4 times higher for walking with the exoskeleton than without, as illustrated for each subject in Fig 2. When averaged over all subjects, oxygen consumption was approximately twice as high when walking in the exoskeleton as without it. The average O_2_ consumption when walking with the exoskeleton (22.5 ± 3.4 ml/min/kg) was also significantly higher (p<0.001) than walking without the exoskeleton (11.7 ± 2.0 ml/min/kg).

**Fig 3:**
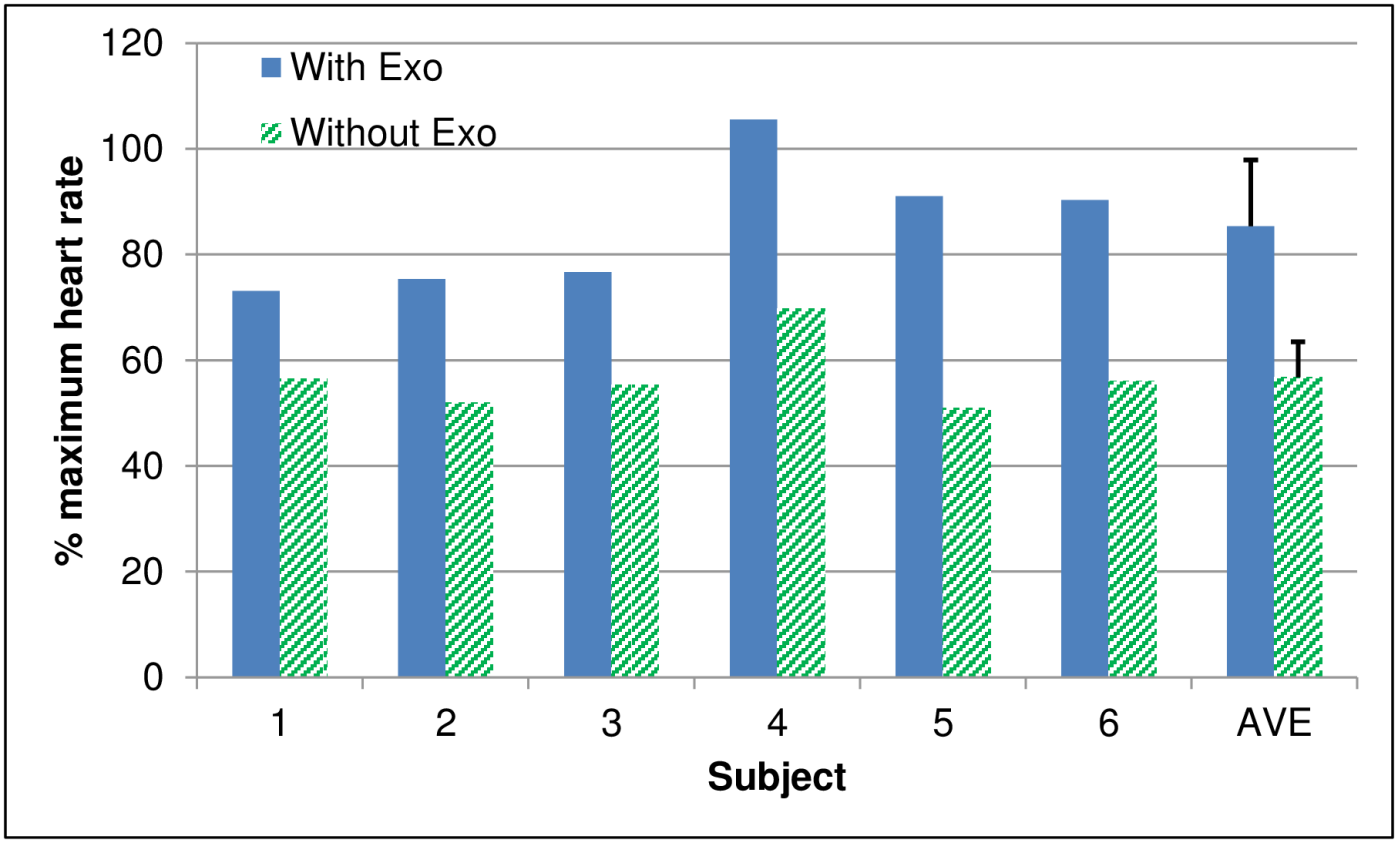
Percent of age corrected maximum heart rate for each subject and averaged across all subjects when walking with and without the exoskeleton.

The average heart rate was at least 30% higher when walking with the exoskeleton than without (Fig 3). The average heart rate ranged from 116 to171 beats/min when walking with the exoskeleton, and from 80 to113 beats/min without the exoskeleton. When corrected for age, percent of the maximum heart rate (i.e. 220 beats/min–age) was significantly higher (p<0.001) when walking with the exoskeleton (85.3 ± 12.6% maximum heart rate) than walking without the exoskeleton (56.7 ± 6.8% maximum heart rate).

The PCI was at least 4.3 times higher when walking with the exoskeleton than without. Average PCI was 1.1 beats/m (range: 0.74 to 1.28 beats/m) when walking with the exoskeleton, and 0.2 beats/m (range: 0.15 to 0.28 beats/m) when walking without the exoskeleton. Similarly, the PCI within each subject was higher when walking with the exoskeleton than walking without the exoskeleton.

## Discussion

The able-bodied participants were able to walk in the exoskeleton at the maximum average speeds of 1.2 m/s. They used an average of 22.5 ± 3.4 ml of O_2_/min/kg (6.5 ± 1.0 METs) which was significantly higher than the average consumption of 11.7 ± 2.0 ml of O_2_/min/kg when walking without the exoskeleton (3.4 ± 0.6 METs). Their heart rate increased significantly when walking with the exoskeleton (148 ± 19 bpm) than walking without (99 ± 14 bpm). It is reasonable to expect that if hybrid neuroprosthesis users with paraplegia were to walk with this exoskeleton at the same speed as participants in this study, their metabolic energy expenditure would be even higher since a much higher effort would be required of upper extremities to maintain balance.

For the sake of comparison, oxygen consumption for normal walking at average speed (1.3 m/s) is about 3.4 METs and subjects with paraplegia walking with bilateral knee-ankle-foot orthoses at 0.28 m/s used an average of 4.6 METs [13]. Similarly, metabolic demand on individuals with paraplegia walking in a powered exoskeleton was approximately 3.3 METs (ranging from 2.2–4.4 METs), walking at 0.22 m/s up to 0.38 m/s with heart rates averaging 118 ± 21 bpm [14,21]. Even if individuals with paraplegia were able to achieve walking speeds for community ambulation with an HNP using this exoskeleton, their rate of oxygen consumption would not be sustainable for practical use over any appreciable distance.

Individuals with paraplegia walking with stimulation alone had PCI values ranging from 2.3 to 6.3 beats/m for walking speeds between 0.2 to 0.4 m/s [10]. An individual with paraplegia walking at 0.42 m/s in a hybrid system combining stimulation with a reciprocal gait orthosis had a PCI of 1.54 beats/m [11]. In our study, able-bodied subjects exhibited a mean PCI of 0.2 beats/m at an average speed of 1.3 m/s, approximating that reported in [12]. The PCI increased from 0.2 to 1.1 beats/m when walking with the exoskeleton and resulted in a less energy efficient gait. While the hybrid system may improve energy cost as measured by PCI when compared to walking with stimulation alone, the PCI would likely increase for individuals with paraplegia walking with this exoskeleton. The various outcome measures of speed, METs, and PCI are summarized in Table 3 for different devices and conditions.

**Table 3:** Speed, METs, and PCI for different conditions and devices.

Device	User	Speed [m/s]	METs	PCI [beats/m]
Exoskeleton	able-bodied	1.2 ± 0.2	6.5 ± 1.0	1.1 (range: 0.74 to 1.28)
Bilateral knee-ankle-foot orthoses [22]	SCI (paraplegia, n = 3)	0.28 ± 0.22	4.6 ± 0.7	--
FNS [10]	SCI (T4-T12)	0.2–0.4 (n = 5)	--	2.3–6.3 (n = 4)
RGO+FNS [11]	SCI (T4, n = 1)	0.42	--	1.54
Powered exoskeletons [14,21]	SCI (C4-L1, n = 116) [14] SCI (T1-T11, n = 8) [21]	0.27 (95% CI: 0.22–0.33) [14] 0.22 ± 0.11 [21]	3.3 (95% CI: 2.2–4.4) [14] 3.2 ± 0.5 [21]	--

(95% CI = 95% confidence interval, C = cervical, L = lumbar, n = sample size, T = thoracic)

The average step length normalized to height was not significantly different within subjects and not significantly different between subjects, except for Subject 5 who had a step length varying from Subjects 1 and 2. Subject 5 had a slightly faster cadence than Subjects 1 and 2, suggesting that Subject 5 may have taken faster but shorter steps. The step length and cadence were most likely at the maxima possible in the exoskeleton due to the constraints of the reciprocally coupled hips, locked ankles, and hydraulic passive resistance.

The average peak hip and knee joint angles of our able-bodied subjects in the exoskeleton were 25° ± 7° and 40° ± 8°, respectively. The peak hip angle was similar to normal peak hip angle (22° ± 5°) during natural cadence (105 ± 6 steps/min), while the peak knee angle was less than normal (65° ± 5°) [23]. The exoskeleton (15.9 kg) adds loads of 4.1 kg below the hip and 2.4 kg below the knee. The added weight of exoskeleton uprights can reduce peak knee joint angles during able-bodied walking [24]. Compensating for the upright weight could enable able-bodied subjects to walk in the exoskeleton without affecting natural gait dynamics. However, adding distal leg loads increases the metabolic rate required for swinging the leg during gait [25].

Average peak hip (128°/sec) and knee (234°/sec) angular velocities for the able-bodied participants walking in the exoskeleton were lower than normal peak hip (~176°/sec) and knee (~343°/sec) angular velocities [23], potentially due to the need to overcome hydraulic passive resistance and device weight. Hydraulic passive resistances at joint angular velocities during walking in the exoskeleton were extrapolated from bench testing data to be 15 Nm at the hip (128°/sec) and 6 Nm at the knee (234°/sec).

FNS is capable of generating about 60 Nm of hip flexion torque and 15 Nm of knee flexion moment [2]. Approximately 25% of the hip flexion torque and 40% of the knee flexion torque generated by FNS would be needed to overcome the passive resistance at the hip and knee joints if walking at similar speeds in this study. This effort would be unlikely sustainable with stimulation for practical walking in persons with paraplegia due to muscle fatigue. Responses to stimulation differ with fatigue and between individuals resulting in varying joint torques and joint angular velocities during stepping.

In this study, the able-bodied participants walked with the knees unlocked in order to eliminate the need for a control system to coordinate joint locking with each step. Locking the knee joints during stance phase could be beneficial for individuals with paraplegia to provide added stability and additional time during gait when the knee extensor muscles could rest to potentially delay the onset of muscle fatigue by reducing duty cycle of muscle activation [16,26].

The exoskeletal component of the HNP, rather than a commercially-available exoskeleton, was selected for this study because its design is compatible with implanted neuroprostheses that individuals with paraplegia would use for stepping [1,2,27]. While the results from this study are mostly applicable to this specific exoskeleton [6,15,16,17,28], the results are also valuable to any hybrid neuroprosthesis exoskeleton design where the primary power for walking is derived from neuromuscular stimulation of paralyzed muscles (i.e. a muscle-first approach) and where exoskeleton constraints, weight, and joints’ passive resistance could place considerable metabolic demand on the user and affect their potential for restoring community level ambulation. This study indicates a need to focus on designing an exoskeleton that reduces the effects of device weight and joints’ passive resistance in order to minimize user effort, unlike the available commercial devices where motive power is derived from motors and the user effort is required only to maintain balance. Future designs of a muscle-first approach should consider external motor power for assistance on an as-needed basis to compensate for weakened muscles and when muscles fatigue with stimulation in order to maintain walking. The passive resistance of exoskeletal joints should be minimized, and actuators used to move the joints should be backdrivable to maximize stimulated walking and physiological benefits of a muscle-first approach.

As commercial technology continues to advance toward incorporating FNS in coordination with the exoskeleton capabilities, there will be a convergence of technologies that will result in their optimal combination into a hybridized system to restore stepping in individuals with paraplegia. The effects of joint constraints, passive resistance and device weight will need to be addressed to achieve this goal. While existing commercially-available powered exoskeletons can successfully restore stepping in individuals with paraplegia, these devices also limit the degrees of freedom to the sagittal plane, have inherent passive resistance in the electric motors, do not provide actuation at the ankle joint, and add weight to each segment of the lower limb, all these factors need to be considered in future system designs that are to be integrated with FNS technology or residual voluntary function.

This study indicates that, even though able-bodied individuals were able to attain community ambulation speeds in the exoskeleton, the metabolic energy expenditure was significantly elevated to levels not sustainable for prolonged walking. This suggests that there are limitations in the system that should be addressed in future designs of devices to restore stepping in individuals with paraplegia. In particular, reducing the passive resistance, having the ability to modulate the reciprocally coupled hips, and unlocking the ankle joint could improve the system. The passive resistance of the hydraulics increased the amount of friction that users had to overcome in order to take each step. Needing to overcome this resistance likely increased the amount of energy that had to be expended. Ideally, the amount of passive resistance should be minimized in any system used to restore gait. By modulating the hip joints between reciprocally coupled and independently freed, the PCI could potentially be lowered by means of increasing step length and speed. However, it is important to balance variable hip coupling with freed hips to ensure that the users have optimal truncal support to reduce forward trunk tilt during walking and reduce the need for upper body support [15]. Activating the paralyzed plantar flexor muscles with neural stimulation could provide the active push-off required to increase gait speed and step length, thus reducing energy cost [29].

Responses to stimulation can differ between each individual with SCI, resulting in different capabilities for restored stepping [1,2]. Muscle fatigue and joint range of motion limitations (e.g., contractures) typical after paralysis may also affect system performance. However, physical therapy and rehabilitation exercises can be designed and implemented for each individual with SCI using stimulation to increase endurance and effectiveness of stimulation for restored stepping [2,27]. Furthermore, constraining hip movements to the sagittal plane with the exoskeleton likely had an effect on speed and efficiency of walking. The weight of the exoskeleton that needs to be overcome during each step should also be minimized.

## Supporting information

S1 Dataset Data used in all analyses. Walking speed, cadence, step length; average METs, heart rate, oxygen consumption, and PCI.
(XLSX)

## References

[1] Kobetic R, Triolo RJ, Marsolais EB. Muscle selection and walking performance of multichannel FES systems for ambulation in paraplegia. IEEE Trans Rehabil Eng. 1997; 5(1):23–29. 9086382 10.1109/86.559346

[2] Kobetic R, Marsolais EB. Synthesis of paraplegic gait with multichannel functional neuromuscular stimulation. IEEE Trans Rehabil Eng. 1994; 2(2):66–79. doi: 10.1109/86.313148

[3] Douglas R, Larson PF, D’Ambrosia R, McCall RE. The LSU reciprocating gait orthosis. Orthopedics. 1983; 6:34–839. doi: 10.3928/0147-7447-19830701-05 24822639 10.3928/0147-7447-19830701-05

[4] Louie DR, Eng JJ, Lam T. Gait speed using powered robotic exoskeletons after spinal cord injury: a systematic review and correlational study. J Neuroeng Rehabil. 2015; 12:82 doi: 10.1186/s12984-015-0074-9 26463355 10.1186/s12984-015-0074-9 PMC4604762

[5] Marsolais EB, Kobetic R, Polando G, Ferguson K, Tashman S, Gaudio R, et al The Case Western Reserve University hybrid gait orthosis. J Spinal Cord Med. 2000; 23(2): 100–108. 10914350 10.1080/10790268.2000.11753516

[6] Kobetic R, To CS, Schnellenberger JR, Audu ML, Bulea TC, Gaudio R, et al Development of hybrid orthosis for standing, walking, and stair climbing after spinal cord injury. J Rehabil Res Dev. 2009; 46(3):447–62. 19675995

[7] Del-Ama AJ, Gil-Agudo A, Pons JL, Moreno JC. Hybrid FES-robot cooperative control of ambulatory gait rehabilitation exoskeleton. J Neuroeng Rehabil. 2014; 11:27 doi: 10.1186/1743-0003-11-27 24594302 10.1186/1743-0003-11-27 PMC3995973

[8] Ha KH, Murray SA, Goldfarb M. An approach for the cooperative control of FES with a powered exoskeleton during level walking for persons with paraplegia. IEEE Trans Neural Syst Rehabil Eng. 2016; 24(4):455–66. doi: 10.1109/TNSRE.2015.2421052 25915961 10.1109/TNSRE.2015.2421052

[9] Gad P, Gerasimenko Y, Zdunowski S, Turner A, Sayenko D, Lu DC, Edgerton VR. Weight bearing over-ground stepping in an exoskeleton with non-invasive spinal cord neuromodulation after motor complete paraplegia. Front Neurosci. 2017; 8;11:333 doi: 10.3389/fnins.2017.00333 28642680 10.3389/fnins.2017.00333 PMC5462970

[10] Winchester P, Carollo JJ, Habasevich R. Physiologic costs of reciprocal gait in FES assisted walking. Paraplegia.1994; 32(10):680–686 doi: 10.1038/sc.1994.110 7831075 10.1038/sc.1994.110

[11] Isakov E, Douglas R, Berns P. Ambulation using the reciprocating gait orthosis and functional electrical stimulation. Paraplegia. 1992; 30(4):239–245. doi: 10.1038/sc.1992.62 1625891 10.1038/sc.1992.62

[12] Ijzerman MJ, Baardman G, van Hof MA, Boom HB, Hermens HJ, Veltink PH. Validity and reproducibility of crutch force and heart rate measurements to assess energy expenditure of paraplegic gait. Arch Phys Med Rehabil. 1999; 80(9):1017–1023. 10489002 10.1016/s0003-9993(99)90054-0

[13] Waters RL, Mulroy S. The energy expenditure of normal and pathologic gait. Gait Posture. 1999;9(3):207–31. 10575082 10.1016/s0966-6362(99)00009-0

[14] Miller ME, Zimmerman AK, Herbert WG. Clinical effectiveness and safety of powered exoskeleton-assisted walking in patients with spinal cord injury: systematic review with meta-analysis. Med Devices. 2016; 9:455–66. doi: 10.2147/MDER.S103102 27042146 10.2147/MDER.S103102 PMC4809334

[15] To CS, Kobetic R, Schnellenberger JR, Audu ML, Triolo RJ. Design of a variable constraint hip mechanism for a hybrid neuroprosthesis to restore gait after spinal cord injury. IEEE/ASME Transactions on Mechatronics. 2008;13(2): 197–205.

[16] To CS, Kobetic R, Bulea TC, Audu ML, Schnellenberger JR, Pinault G, et al Stance control knee mechanism for lower-limb support in hybrid neuroprosthesis. J Rehabil Res Dev. 2011; 48(7):839–50. 21938668 10.1682/jrrd.2010.07.0135 PMC4030381

[17] To CS, Kobetic R, Bulea TC, Audu ML, Schnellenberger J, Pinault GC, et al Sensor-based hip control with a hybrid neuroprosthesis for walking in paraplegia. J Rehabil Res Dev. 2014; 51(2):229–244. doi: 10.1682/JRRD.2012.10.0190 24933721 10.1682/JRRD.2012.10.0190

[18] Perry J, Garrett M, Gronley JK, Mulroy SJ. Classification of walking handicap in the stroke population. Stroke. 1995; 26(6):982–989. 7762050 10.1161/01.str.26.6.982

[19] Lapointe R, Lajoie Y, Serresse O, Barbeau H. Functional community ambulation requirements in incomplete spinal cord injured subjects. Spinal Cord. 2001; 39(6):327–335. doi: 10.1038/sj.sc.3101167 11438855 10.1038/sj.sc.3101167

[20] Robinett CS, Vondran MA. Functional ambulation velocity and distance requirements in rural and urban communities. A clinical report. Phys Ther. 1988;68(9):1371–1373. 3420171 10.1093/ptj/68.9.1371

[21] Asselin A, Knezevic S, Kornfeld S, Cirnigliaro C, Agranova-Breyter I, Bauman WA, et al Heart rate and oxygen demand of powered exoskeleton-assisted walking in persons with paraplegia. J Rehabil Res Develop. 2015; 52(2):147–158. doi: 10.1682/JRRD.2014.02.0060 26230182 10.1682/JRRD.2014.02.0060

[22] Waters RL, Lunsford BR. Energy cost of paraplegic locomotion. J Bone Joint Surg Am. 1985; 67(8):1245–50. 4055849

[23] Winter DA. The biomechanics and motor control of human gait: normal, elderly and pathological. 2nd ed University of Waterloo, 1991.

[24] Murray S, Goldfarb M. Towards the use of a lower limb exoskeleton for locomotion assistance in individuals with neuromuscular locomotor deficits. Conf Proc IEEE Eng Med Biol Soc. 2012; 1912–1915, 2012 doi: 10.1109/EMBC.2012.6346327 23366288 10.1109/EMBC.2012.6346327 PMC3688043

[25] Browning RC, Modica JR, Kram R, Goswami A. The effects of adding mass to the legs on the energetics and biomechanics of walking. Med Sci Sports Exerc. 2007; 39(3):515–525. doi: 10.1249/mss.0b013e31802b3562 17473778 10.1249/mss.0b013e31802b3562

[26] Kobetic R, Marsolais EB, Semame P, Borges G. Human Walking. 2nd ed Rose J, Gamble JG, editors. Baltimore, Maryland, USA: Williams & Wilkins; 1994 Chapter 10, The Next Step: Artificial Walking; p.230.

[27] Triolo R, Chang S, Kobetic R. Functional electrical stimulation and spinal cord injury in Lower Extremity Functional Restoration with FES. Physical Medicine and Rehabilitation Clinics of North America: Spinal Cord Injury Rehabilitation, Ho C., editor. Elsevier, Philadelphia, Pennsylvania. 2014;25(3):631–654. 10.1016/j.pmr.2014.05.001 PMC4519233 25064792

[28] Chang SR, Nandor MJ, Lu L, Kobetic R, Foglyano KM, Schnellenberger JR, Audu ML, Pinault G, Quinn RD, Triolo RJ. A muscle-driven approach to restore stepping with an exoskeleton for individuals with paraplegia. J Neuroeng Rehabil. 2017; 14(1):48 doi: 10.1186/s12984-017-0258-6 28558835 10.1186/s12984-017-0258-6 PMC5450339

[29] Mooney LM, Rouse EJ, Herr HM. Autonomous exoskeleton reduces metabolic cost of human walking. J NeuroEng Rehabil. 2014; 11:151 doi: 10.1186/1743-0003-11-151 25367552 10.1186/1743-0003-11-151 PMC4236484

